# Parental Decision-Making for Themselves and Their Children in a Metropolis of China: Comparing Influenza and Rotavirus Vaccination Under the Behavioral and Social Drivers Framework

**DOI:** 10.3390/vaccines14040340

**Published:** 2026-04-12

**Authors:** Yilan Xia, Jie Fei, Xiangting Zhang, Peisong Zhong, Yihan Lu, Qian Zhang

**Affiliations:** 1 Shanghai Institute of Infectious Disease and Biosecurity, Fudan University, Shanghai 200032, China; ylxia24@m.fudan.edu.cn (Y.X.); xiangtingzhang25@m.fudan.edu.cn (X.Z.); 2 Department of Epidemiology, Ministry of Education Key Laboratory of Public Health Safety, School of Public Health, Fudan University, Shanghai 200032, China; 3 Department of Immunization Planning, Shanghai Jiading District Center for Disease Control and Prevention, Shanghai 201800, China; feijie@jiading.gov.cn (J.F.); zhongps@126.com (P.Z.)

**Keywords:** behavioral and social drivers, influenza vaccine, rotavirus vaccine, parental decision-making, vaccine motivation, vaccine hesitancy

## Abstract

**Background**: Parents serve as the primary decision-makers for childhood vaccination while also making decisions regarding their own vaccination, yet vaccination decision drivers are typically studied separately by vaccine type or target population. **Methods**: This study investigated parental decision-making processes for two self-paid and non-National Immunization Program vaccines in China, childhood rotavirus vaccine and adult influenza vaccine, by utilizing a structured survey grounded in the World Health Organization Behavioral and Social Drivers of Vaccination framework. Spearman’s rank correlation coefficients were used to assess the consistency of parental attitudes toward the two vaccines across behavioral and social driver domains. Structural equation models were conducted separately for childhood and adult vaccines to examine decision-making pathways. **Results**: The findings indicated that parental drivers related to awareness, social processes, and practical issues showed a high consistency across adult and childhood vaccination decisions (r > 0.7), whereas the consistency in vaccination behaviors remained low (r = 0.21). Compared with adult vaccination, childhood vaccination decisions were more strongly influenced by vaccine safety concerns and healthcare practitioners’ recommendations, which emerged as key drivers. Furthermore, family norms emerged as an effectively shared driver of vaccination decisions for both adult and childhood vaccines (adult: *β* = 0.784; childhood: *β* = 0.970). **Conclusions**: By jointly synthesizing adult and childhood vaccination decisions from a parental perspective, this study provides crucial evidence to support the development of integrated, family-centered strategies to improve vaccine uptake.

## 1. Introduction

Rotavirus vaccines have been introduced into national immunization programs (NIP) in more than 120 countries worldwide and have substantially reduced rotavirus-associated morbidity and mortality in both high-income and low- and middle-income settings [1]. In China, real-world evidence has also demonstrated high vaccine effectiveness, with the pentavalent rotavirus vaccine showing up to 82.7% effectiveness against rotavirus gastroenteritis after three doses [2]. In addition, population-based studies indicate that higher vaccine coverage is associated with a 32.4% reduction in rotavirus gastroenteritis incidence among children under 5 years of age, along with indirect protection among unvaccinated children through herd immunity [3]. Four rotavirus vaccines are licensed in China: the monovalent LLR (in use since 2000), the imported pentavalent RV5 (since 2018), the recently approved LLR3 (2023) and RV6 (2025), all of which have maintained favorable safety profiles [4]. However, primarily due to national budgetary constraints [5], rotavirus vaccines remain classified as non-National Immunization Program (non-NIP) vaccines, and their uptake remains relatively low. In 2019, the three-dose coverage rate of rotavirus vaccination in 10 provinces in China was estimated to be only 1.8% [6], which was considerably lower than that of other non-NIP vaccines recommended for children of the same age, such as haemophilus influenzae type b (Hib) and pneumococcal conjugate vaccines [7]. Three types of influenza vaccines are available in China, and these vaccines have been shown to reduce the risk of severe illness, particularly among high-risk populations, and are considered cost-effective in China [8,9]. However, publicly funded vaccination programs for priority groups, such as older adults and children, are limited to a few regions. Similarly, influenza vaccine uptake among adults in China also remains low. During the 2021/22 influenza season, the coverage rate of self-paid influenza vaccination in China was approximately 1.81% [10]. Therefore, it is crucial to improve the uptake of rotavirus vaccination in children and influenza vaccination in adults. However, vaccination settings and target populations for these two vaccines differ substantially.

Vaccination represents a typical behavioral decision-making process that is jointly influenced by multiple factors, including individual awareness, social influences, and practical accessibility. The World Health Organization (WHO) Behavioral and Social Drivers (BeSD) of Vaccination framework provides a theoretical tool for systematically characterizing these factors [11]. For children under 4 years of age, vaccination decisions rely entirely on parents as the primary decision-makers. Therefore, rotavirus vaccine uptake among young children largely reflects parental vaccine decision-making behaviors. Previous studies on childhood vaccination decision-making have predominantly adopted quantitative, qualitative, or mixed-methods approaches, with a primary focus on parental attitudes toward childhood vaccines [12,13,14]. A study conducted in 2022 across three cities in China found that, when deciding whether to vaccinate their children against rotavirus, parents showed a stronger preference for vaccines with fewer side effects, higher effectiveness, longer protection duration and lower cost, by using discrete choice experiment [15]. In contrast, a cross-sectional study conducted in 2013 among parents of children under 3 years of age examined determinants of rotavirus vaccination and found that parental concerns regarding vaccine cost, needle pain, and safety did not have a significant impact on vaccine uptake, whereas the frequency of recommendations from healthcare practitioners’ providers and parental health literacy were significantly associated with vaccine uptake [16].

Existing evidence from studies on human papillomavirus (HPV) and COVID-19 vaccines suggests a significant positive association between parents’ own vaccination behaviors and the vaccination decisions they make for their children [17,18], with certain shared drivers, such as vaccine confidence and cost [19]. Furthermore, parents tend to exhibit greater caution when making vaccination decisions for their children than when deciding on their own vaccination, as documented in influenza vaccination in the United Kingdom [20]. However, previous studies have rarely conducted systematic comparison of behavioral drivers underlying parental decisions regarding parental and childhood vaccination. It remains limited to comprehensively analyze the similarities and differences between these two types of vaccination decisions within the BeSD framework. In public health practice, vaccine communication, health education, and social mobilization in vaccination campaigns are typically implemented at the population level. Therefore, it may be both effective and feasible to improve vaccination strategies of multiple vaccines that share common drivers. The hypothesis of this study was that parental decision-making regarding adult vaccination and childhood vaccination differs in key behavioral and social factors, leading to potentially distinct uptake across these two vaccine types. At the same time, our study aimed to identify shared behavioral and social drivers underlying parental decisions regarding parental and childhood vaccination, which provided a more comprehensive understanding to support the development of more integrated vaccination strategies.

## 2. Materials and Methods

### 2.1. Study Design

To systematically compare the structure of parental vaccination decision-making across different target populations, this study focused on two self-paid and non-NIP vaccines in China: influenza vaccine for parents and rotavirus vaccine for their children. Both vaccines currently have relatively low coverage rates in China. Vaccination decisions for both vaccines are primarily based on individual or household choice and are not accompanied by nationwide mandatory policy requirements or economic compensation measures. These characteristics help to minimize the potential influence of policy-related disparity on vaccination decision-making within a unified analytical framework and enhance the comparability across different decision contexts.

This study employed a cross-sectional study design to investigate the determinants of vaccine uptake among parents in Jiading District, Shanghai, during July–August 2025. An anonymous questionnaire survey was administered to parents of children under 4 years of age who visited vaccination clinics for vaccination or consultation. We excluded respondents who were not the child’s guardian or primary caregiver, as well as those who submitted incomplete or inconsistent questionnaires. All respondents were required to complete survey items regarding both their own influenza vaccination decisions and their decisions regarding rotavirus vaccination for their children.

### 2.2. Questionnaire Design and Measurement

The questionnaire was developed specifically for this study and has not been previously published. We prepared an expert panel, including healthcare practitioners from centers for disease control and prevention (CDCs) and points of vaccination (POVs), and parents of young children, to develop and finalize the survey questionnaire, based on a BeSD questionnaire designed for rotavirus vaccine [21]. The English version of the full questionnaire is provided in Supplementary File.

The questionnaire consisted of five components: (1) sociodemographic characteristics of parents, including birth year, sex, relationship to child, educational levels, annual household income, and marital status; (2) parental knowledge of rotavirus and rotavirus vaccines; (3) BeSD items of rotavirus vaccination in children; (4) parental knowledge of influenza and influenza vaccines; and (5) BeSD items of influenza vaccination among parents. The knowledge scores were assessed using single-choice questions, with one point assigned for each correct response. Total knowledge scores were calculated by summing the item scores, with higher scores indicating greater knowledge. To enhance the comparability of vaccination decision-making between adult and childhood vaccines, the questionnaire design sought to maintain consistency in question constructs and wording across the two vaccines. For descriptive analyses, BeSD variables were subsequently collapsed into binary categories (agree vs. disagree) to facilitate interpretation of response patterns. In the SEM analyses, the original ordinal response scales were retained and modeled as ordered categorical variables. The BeSD questionnaire items for childhood rotavirus vaccination and adult influenza vaccination are presented in Table 1.

### 2.3. Questionnaire Distribution

From July through August 2025, parents of children under 4 years of age visiting 17 community vaccination clinics in Jiading District, Shanghai, were recruited using a convenience sampling strategy. Participants completed an online questionnaire via scanning a QR code powered by www.wjx.com. In our study, the child under 4 years of age was defined as the youngest child in the family. The required sample size for the survey was estimated using the formula: $n=\frac{Z_{\alpha /2}^{2}}{d^{2}}*p*\left(1-p\right)$. Based on the 2019 Shanghai data, at least one-dose coverage of rotavirus vaccine was 47.0% and the full-course coverage was 11.4% [6]. The sample size was calculated with a significance level (α) of 0.05 (corresponding to $Z_{\alpha /2}=1.96$) and a relative margin of error of 20% (d=0.20∗p), based on a full-course coverage rate of 11.4% (*p* = 0.114), resulting in a calculated sample size of 747. To account for potential invalid responses, the target sample size was increased by 20%, yielding a final target of 926 participants.

### 2.4. Statistical Analysis

Continuous variables were summarized as means ± standard deviations, and categorical variables as frequencies and proportions. Skewed distributions of knowledge scores were described using medians and interquartile ranges (IQRs). Variables associated with vaccine uptake were analyzed using Pearson’s chi-square test or Fisher’s exact test, as appropriate. The normality of knowledge score distributions was assessed using the Shapiro–Wilk test, and differences in knowledge score accuracy between the two vaccines were evaluated using the Mann–Whitney U test.

A structural equation model (SEM) was employed to characterize latent psychosocial constructs and their interrelationships, enabling a comparison of parental decision-making mechanisms for their own vaccination versus childhood vaccination within the BeSD framework. Thinking and Feeling was modeled as an upstream construct influencing Social Processes and Practical Issues, which in turn predicted vaccination behavior. Sociodemographic variables were included as exogenous predictors of Thinking and Feeling. The model structure was informed by the prior literature [22]. Prior to building the full structural model, confirmatory factor analysis (CFA) was conducted to examine the measurement validity of observed variables for the BeSD constructs. Variables with factor loadings <0.40 were considered insufficiently representative and were excluded from subsequent analyses, consistent with commonly accepted standards for interpretability in factor analysis [22]. The SEM was then developed to assess the path relationships among BeSD variables. Disease and vaccine-related knowledge items were incorporated into the *Thinking and feeling* latent construct for modeling. Sociodemographic variables that were significantly associated with vaccination decisions in univariate analyses (*p* < 0.001) were included in the model as covariates. All BeSD variables were treated as ordered categorical variables in the SEM and analyzed using a diagonally weighted least squares (DWLS) estimator.

To evaluate correlations between BeSD domains regarding personal influenza vaccination and childhood rotavirus vaccination, pairwise correlations were calculated across the four domains of the BeSD framework. Spearman’s rank correlation coefficients were used for these analyses, based on individual-level domain scores.

Data were organized and managed in Microsoft Excel 2016 (Microsoft Corporation, Redmond, WA, USA). All statistical analyses and models were implemented using (version 4.5.0; R Foundation for Statistical Computing, Vienna, Austria).

## 3. Results

### 3.1. Basic Characteristics of Respondents

A total of 951 valid questionnaires were included in the final analysis, following review and exclusion of questionnaires with logical inconsistencies or missing responses. The Cronbach’s α value for all variables was 0.76. The Kaiser–Meyer–Olkin (KMO) value of the questionnaire was 0.83 (Bartlett’s test of sphericity, χ^2^ = 2845.66, *p* < 0.001). The mean age of respondents was 31.33 ± 5.38 years. Of them, 74.5% were female, 78.5% had attained a bachelor’s degree or above, and 71.7% were mothers of the children (Table 2). Regarding rotavirus vaccination status of the youngest child in the family, 24.4% had never received rotavirus vaccination, 36.4% had received partial doses with the next dose not yet due, 2.4% had received partial doses but did not intend to complete the remaining schedule, and 36.8% had completed the full vaccination schedule. Educational level and annual household income were strongly associated with childhood rotavirus vaccination (each *p* < 0.001) (Table 2). Furthermore, 39.3% of respondents reported having received the 2024/25 seasonal influenza vaccine. Educational level was associated with parental influenza vaccination (*p* = 0.026) (Table 2).

### 3.2. Knowledge of Rotavirus Vaccine and Influenza Vaccine

Respondents correct response rates of knowledge items for rotavirus and rotavirus vaccine ranged from 54.4% to 88.5%. The median knowledge score was 7 points (IQR: 5–8). High correct response rates were observed for knowledge regarding general awareness of rotavirus vaccine (88.5%), optimal prevention strategies (83.6%), high-risk populations for rotavirus infection (77.5%), clinical manifestations (75.1%), and vaccine effectiveness (70.2%) (Table 3).

For influenza and influenza vaccine-related knowledge, correct response rates across the seven knowledge items ranged from 50.0% to 92.4%, with a median score of 6 points (IQR: 5–7) (Table 3). Similarly, high correct response rates were observed for general awareness of influenza vaccine (92.4%) and high-risk populations for complications (88.4%). In contrast, lower knowledge levels were noted for the distinction between influenza and common cold (50.0%) and the necessity of annual influenza vaccination (63.6%), indicating persistent gaps in understanding characteristics of influenza and influenza vaccination. It did not differ significantly between parental knowledge scores for rotavirus and influenza vaccination (*p* = 0.887).

Knowledge of the vaccines and vaccination was significantly associated with vaccination status, regardless of influenza vaccination in parents or rotavirus vaccination in their children (each *p* < 0.001). Higher knowledge scores were observed more frequently among respondents with both childhood rotavirus vaccination (*p* < 0.001) and 2024/25 influenza vaccination (*p* < 0.001) (Table 2).

### 3.3. Characterization of BeSD Domains and Responses

**Figure 1:**
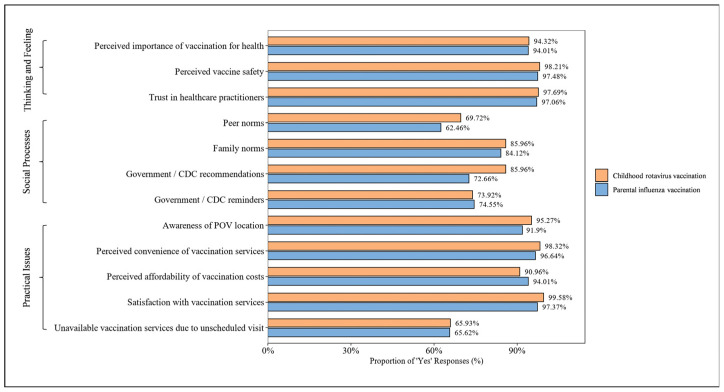
Parental responses to children’s rotavirus vaccination and their own influenza vaccination by the Behavioral and Social Drivers (BeSD) of Vaccination domains. Proportion of respondents reporting ‘Yes’ for each item in the BeSD framework, comparing parental responses regarding children’s rotavirus vaccination and their own influenza vaccination. CDC, center for disease control and prevention; POV, point of vaccination.

Under the BeSD framework, within the *Thinking and feeling* domain, 94.3% of respondents perceived rotavirus vaccination as important for their children’s health, 98.2% considered rotavirus vaccination to be safe for children, and 97.7% reported trust in healthcare practitioners (Figure 1). Within the *Social processes* domain, 69.7% of respondents reported childhood rotavirus vaccination was influenced by peer norms (i.e., vaccination behaviors of people around them), 85.0% by family members, and 73.9% by reminders from government/CDC (Figure 1). Within the *Practical issues* domain, 95.3% of respondents were aware of POV locations, 98.3% perceived the convenience of vaccination services, 91.0% perceived the affordability of vaccination costs, and 99.6% were satisfied with vaccination services.

All variables within the *Thinking and feeling* domain were significantly associated with childhood rotavirus vaccination (each *p* < 0.001). Within the *Social processes* domain, recommendations from government/CDC (χ^2^ = 0.20, *p* = 0.655) was not significantly associated with rotavirus vaccination. Within the *Practical issues* domain, unavailable vaccination due to unscheduled visit was not significantly associated with rotavirus vaccination (χ^2^ = 3.63, *p* = 0.057).

Consistent with findings for childhood rotavirus vaccination, parents generally exhibited a highly positive attitude toward influenza vaccination, particularly within the *Thinking and feeling* domain (Figure 1). In contrast to childhood rotavirus vaccination, differences were observed in the level of social and family support for parental influenza vaccination. The 62.5% of respondents reported their influenza vaccination was influenced by peer norms, which was lower than that reported in their children’s rotavirus vaccination (69.7%) (χ^2^ = 46.707, *p* < 0.001). In addition, 72.7% was influenced by recommendations from government/CDC in their influenza vaccination, compared with 86.0% in their children’s rotavirus vaccination (χ^2^ = 101.17, *p* < 0.001).

Unlike childhood rotavirus vaccination, within the *Thinking and feeling* domain, only perceived importance of vaccination for health was significantly associated with parental influenza vaccination (*p* < 0.001). Within the *Social processes* domain, recommendations from government/CDC (*p* = 0.001) and peer norms (*p* = 0.001) were significantly associated with parental influenza vaccination. Within the *Practical issues* domain, perceived convenience of influenza vaccination (*p* = 0.012) and perceived affordability of influenza vaccination (*p* < 0.001) were significantly associated with parental influenza vaccination.

Moreover, correlations were generally high between BeSD domains of influenza vaccination in parents and rotavirus vaccination in children, especially in *Thinking and feelings* (r = 0.82), *Social processes* (r = 0.71), and *Practical issues* (r = 0.84) (Table 4). Parental awareness, social influences, and practical accessibility regarding their own influenza vaccination were largely consistent with their attitudes toward rotavirus vaccination for their children. However, parental influenza vaccine uptake was weakly correlated with their children’s rotavirus vaccine uptake (r = 0.21).

### 3.4. Correlations Between BeSD Domains

SEM was applied to determine the relationships among three latent constructs (*Thinking and feeling*, *Social processes*, and *Practical issues*) for rotavirus vaccination in children and influenza vaccination in parents. *Motivation* was measured using a single item and was therefore included in the models as an observed variable.

In the CFA for the rotavirus vaccination model, no indicator exhibited a factor loading below 0.4. For the influenza vaccination model, two indicators (government/CDC recommendations and unavailable vaccination services due to unscheduled visit) were excluded due to factor loadings below 0.4. To ensure structural comparability between the two SEMs, these two indicators were subsequently removed from both models in the final analyses. The final rotavirus vaccination SEM demonstrated adequate fit (CFI = 0.982, TLI = 0.975, SRMR = 0.070, RMSEA = 0.053; 90% CI, 0.044–0.062). The influenza vaccination SEM also showed good model fit, with CFI = 0.991, TLI = 0.987, SRMR = 0.044, RMSEA = 0.028 (90% CI, 0.017–0.039).

**Figure 2:**
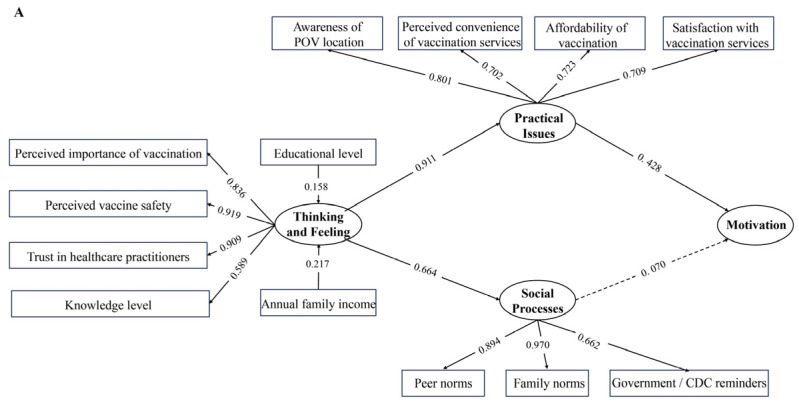

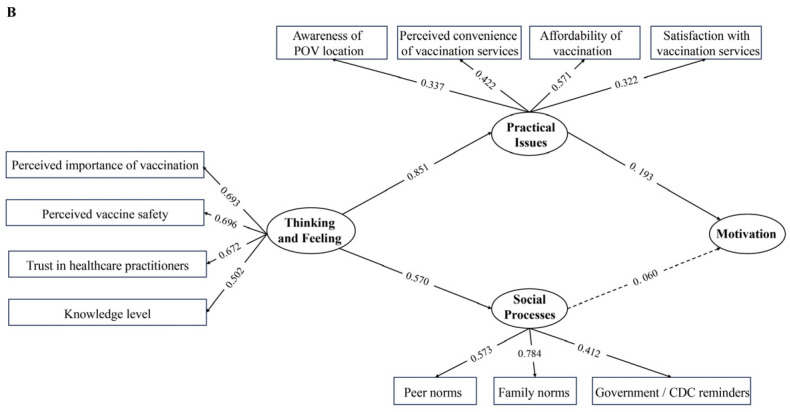
Structural equation models of the Behavioral and Social Drivers of Vaccination domains for childhood rotavirus vaccination (**A**) and parental influenza vaccination (**B**). Numbers above the arrows represent standardized regression coefficients (*β*) obtained from the structural equation model. Dashed rows indicate non-significant paths; solid rows indicate statistically significant paths.

For rotavirus vaccination, with the exception of the path from *Social processes* to *Motivation*, all specified relationships between latent constructs and their observed indicators were statistically significant (Figure 2). *Thinking and feeling* showed strong positive associations with *Social processes* (*β* = 0.664, Z = 16.05, *p* < 0.001) and *Practical issues* (*β* = 0.911, Z = 11.21, *p* < 0.001). *Practical issues* were positively associated with *Motivation* (*β* = 0.428, Z = 7.41, *p* < 0.001). In addition, higher annual household income (*β* = 0.217, Z = 5.10, *p* < 0.001) and higher educational levels (*β* = 0.158, Z = 3.90, *p* < 0.001) were positively associated with *Thinking and feeling*.

For influenza vaccination, *Thinking and feeling* were positively associated with *Social processes* (*β* = 0.570, Z = 11.461, *p* < 0.001) and *Practical issues* (*β* = 0.851, Z = 9.982, *p* < 0.001) (Figure 2). *Practical issues* showed a weaker but statistically significant association with *Motivation* (*β* = 0.193, Z = 4.935, *p* < 0.001), whereas *Social processes* were not significantly associated with *Motivation* (*β* = 0.060, Z = 1.311, *p* = 0.190).

Standardized factor loadings for all latent constructs were consistently higher in the childhood rotavirus vaccination model than in the parental influenza vaccination model. Within the *Thinking and feeling*, perceived vaccine safety and trust in healthcare practitioners were the most representative indicators (rotavirus vaccination model, *β* = 0.919, *β* = 0.909; influenza vaccination model, *β* = 0.696, *β* = 0.672). Family norms exhibited the highest loading within the *Social processes* construct (rotavirus vaccination model, *β* = 0.970; influenza vaccination model, *β* = 0.784). Within the *Practical issues* construct, POV location and satisfaction with vaccination services showed relatively higher loadings in the rotavirus vaccination model.

## 4. Discussion

Both rotavirus vaccine and influenza vaccine have well-established public health value in controlling disease transmission and reducing disease-related outpatient visits and hospitalizations [23,24]. However, previous studies across multiple vaccines have repeatedly shown that positive vaccine attitudes formed at the individual or parental level often translate inefficiently into actual vaccine uptake [12,25,26,27]. By situating parental decisions regarding adult and childhood vaccination within a unified analytical framework, this study systematically compared similarities and differences in vaccination decision-making mechanisms across the two vaccine contexts. Our findings indicated that parents exhibited highly consistent attitudinal structures across awareness, social influences, and practical accessibility domains (r > 0.7), whereas substantial disparity was observed at the level of final vaccination behaviors between rotavirus vaccination in children and influenza vaccination in parents (r = 0.21). Additionally, our study identified several policy-relevant clues operating across both adult and childhood vaccination decisions, providing empirical support for improving the non-NIP vaccine uptake at the household level.

To further explore the underlying motivations for vaccination, our study employed SEM within a unified theoretical framework to compare the determinants of parental decisions regarding adult vaccination versus childhood vaccination. In the childhood rotavirus vaccination context, parental *Thinking and feeling* were more tightly linked to *Social processes* and *Practical issues*. Previous studies have indicated that, compared with individuals making vaccination decisions for themselves, parents tended to be more sensitive to potential negative consequences when deciding vaccination for their children [28]. A U.S. study published in 2025 reported that parents exhibited heightened affective risk perception in HPV vaccination decisions for their children, and their knowledge was negatively associated with vaccine intent; in contrast, among young adults making vaccination decisions for themselves, knowledge exerted a positive effect [29]. Our findings found for both childhood rotavirus vaccination and parental influenza vaccination, disease- and vaccine-related knowledge did not constitute the primary drivers within the *Thinking and feelings* construct (*β* = 0.589 and 0.502, respectively). Nevertheless, knowledge remained significantly and positively associated with final vaccination decisions (*p* < 0.001). This suggests that knowledge may function as a distal or enabling factor by providing the cognitive basis for vaccine acceptance, rather than acting as a proximal driver that directly precipitates the behavior. In the context of information saturation, simply increasing knowledge may be insufficient to bridge the gap between understanding and behavioral change, particularly regarding childhood vaccination. For HPV vaccination, comprehensive information disclosure helped parents provide evidence for decision-making [30]. Meanwhile, in an Italian Twitter-based study, governmental positions and public policy signals exerted a stronger influence on parental vaccine hesitancy than vaccine attributes [31]. Thus, our study suggests that determining the drivers that facilitate the effective translation of positive vaccine attitudes into actual uptake remains a complex challenge. Moreover, an optimal strategy may involve considering distinct vaccination motivations and implementing tailored interventions across vaccines and populations.

Our study further demonstrated that parents relied more heavily on perceived vaccine safety and trust in the healthcare practitioners (*β* > 0.9) in the context of childhood vaccination decisions, compared with decisions for their own adult vaccination (*β* < 0.7). Previous studies have suggested that trust in the healthcare practitioners is largely shaped by prior healthcare experiences and exposure to information; however, once distrust is formed following adverse events, it may persist even after the events have been resolved [32]. In the childhood vaccination setting, such trust appears to be particularly critical. Concerns regarding healthcare practitioners and vaccine safety have consistently been identified as key determinants of parental vaccine hesitancy [15,21,33]. A study conducted in China in 2013 showed that the frequency of recommendations from physicians at vaccination clinics was strongly associated with rotavirus vaccine uptake (OR = 3.56, 95% CI, 2.20–5.75), with an effect size exceeding that observed for Hib and varicella vaccines [16]. Similarly, a Swiss study published in 2018 reported that healthcare practitioners remained the most trusted source of vaccine-related information among parents, regardless of whether they exhibited vaccine hesitancy toward childhood immunization [34]. Therefore, in childhood vaccination decision-making, leveraging information delivery by healthcare practitioners may strengthen parental trust in vaccine safety and serve as a crucial strategy for increasing vaccine uptake.

Based on the BeSD framework and focusing on low- and middle-income countries, a literature review published in 2024 proposed five policy directions to improve vaccine uptake, including social groups and community mechanisms, setting expectations and communicating the role of providers in immunization, strengthening health system factors holistically, mitigating the indirect costs, and plan for variability in demand for vaccination [35]. Consistent with these recommendations, our findings from a large urban setting in China supported the applicability of BeSD-informed policy strategies in the context of childhood vaccination. Furthermore, we found that parental educational level was significantly associated with the uptake of adult and childhood vaccines. Specifically, parents with lower educational level tend to have lower health literacy [36] limiting their capacity to critically assess misinformation [37], which may hinder vaccine uptake for both themselves and their children. Therefore, our findings further suggest that families with lower income and lower educational attainment may require additional, targeted health education interventions [38].

In both adult and childhood vaccination analyses, family norms exerted a significant positive influence within the *Social processes* domain for both vaccines (adults: *β* = 0.784; children: *β* = 0.970), in line with prior evidence [39,40]. However, emerging studies suggests that the effects of family interactions may vary by communication patterns and social dynamics. A study conducted in 2020 among African American populations reported that open-ended vaccine discussions could paradoxically reinforce negative parental beliefs toward childhood vaccination [39]. In contrast, a nationally representative U.S. study published in 2022 demonstrated that distributing conversation cards to vaccinated individuals facilitated communication with unvaccinated family members and friends, which reduced COVID-19 vaccine hesitancy [41]. These findings suggest that positive parental guidance and role modeling in the family have the potential to simultaneously improve vaccine uptake in both adults and children. It warrants further clarifying the potential influence by public health measures and interventions across multiple vaccination scenarios.

Overall, our findings identify both shared and distinct behavioral and social factors underlying parental decision-making regarding adult versus childhood vaccination. Decisions for childhood vaccination were more strongly influenced by safety concerns and reliance on healthcare provider recommendations. In line with previous studies, current non-NIP strategies in China have largely focused on health education, yet their effectiveness may be limited by misinformation and insufficient tailoring for populations with lower educational attainment [7]. Our findings suggest that, beyond general awareness campaigns, targeted interventions for parents in low-education families are essential, focusing on correcting vaccine-related misinformation and leveraging healthcare provider recommendations to effectively promote the uptake of non-NIP vaccines.

Our study emphasized whether the decision-making mechanisms of the same individual remain consistent across two distinct contexts: self-vaccination and childhood vaccination, showing the study strength. Also, this study has several limitations. First, our sample primarily consisted of female caregivers. Previous study indicated significant disparity between mothers and fathers in vaccination concerns [42], with mothers being disproportionately more likely to exhibit vaccine hesitancy [43]. Second, the BeSD framework did not explicitly incorporate the influence of social media, although the impact of platforms like WeChat (a Chinese counterpart of WhatsApp) may be manifested through peer norms. A systematic review indicated that vaccines are the most prevalent topic of health misinformation on social media [44], with misleading content generating significantly higher levels of engagement than evidence-based scientific information [45,46]. Misinformation and disinformation have emerged as primary determinants of health perceptions and behaviors during the COVID-19 pandemic, while also fostered distrust regarding vaccines among certain groups [12,47]. Although the respondents mostly exhibited good health literacy, it remains necessary to evaluate the impact of eHealth and media literacies on online health information [48]. Third, childhood rotavirus and parental influenza vaccines differed in the contexts, which resulted in potentially original disparity in the drivers to vaccine uptake. Fourth, as this study was conducted in a major urban center among voluntary participants, the findings may overestimate positive vaccine attitudes and trust due to self-selection bias; thus, the results may not be generalizable to rural populations or highly vaccine-hesitant groups.

## 5. Conclusions

This study examined parental decision-making regarding adult influenza vaccination and childhood rotavirus vaccination from the caregiver perspective. We found that parents exhibited a highly consistent structure of awareness, social influences, and practical accessibility across the two vaccination contexts, whereas substantial disparity emerged at the level of vaccination behaviors, with decisions for childhood vaccination being more strongly shaped by risk perception. Moreover, family norms played a crucial role in both adult and childhood vaccination, suggesting that family-based guidance and role modeling may concurrently improve the self-paid vaccine uptake among adults and children. Our study provided essential data and evidence for future vaccination promotion strategies.

## Figures and Tables

**Table 1:** Items assessing behavioral and social drivers of rotavirus vaccination in children and influenza vaccination in parents.

	Rotavirus Vaccination	Influenza Vaccination
Thinking and Feeling	Perceived importance of rotavirus vaccination for children’s health	Perceived importance of influenza vaccination for personal health
	Perceived safety of rotavirus vaccination for children	Perceived safety of influenza vaccination for adults
	Trust in healthcare practitioners administering rotavirus vaccination to children	Trust in healthcare practitioners administering influenza vaccination to adults
Social Processes	Perceived support from family for vaccinating children with rotavirus vaccine	Perceived support from family for receiving influenza vaccine
	Advice from relatives, friends, or colleagues to vaccinate children with rotavirus vaccine	Advice from relatives, friends, or colleagues to receive influenza vaccine
	Recommendation from government/CDC to vaccinate children with rotavirus vaccine	Recommendation from government/CDC to receive influenza vaccine
	Reminders from government/CDC regarding children reaching the recommended age for rotavirus vaccination	Reminders from government/CDC regarding influenza vaccination during the flu season
Practical Issues	Awareness of rotavirus vaccination sites	Awareness of influenza vaccination sites
	Unavailable vaccination services for children due to unscheduled visit	Unavailable vaccination services due to unscheduled visit
	Convenience of rotavirus vaccination for children	Convenience of influenza vaccination for adults
	Ability to pay the cost of rotavirus vaccination, including vaccine price and transportation fee	Ability to pay the cost of influenza vaccination, including vaccine price and transportation fee
	Satisfaction with rotavirus vaccination services for children	Satisfaction with influenza vaccination services for adults
Motivation	Rotavirus vaccine uptake in children	Influenza vaccine uptake in adults during 2024/25 flu season

**Table 2:** Variables associated with rotavirus vaccination in children and 2024/25 influenza vaccination in parents.

Characteristics	Total (N = 951)		Childhood Rotavirus Vaccination §				Parental Influenza Vaccination (2024/25 Season)			
	n	%	n	%	χ2	*p* Value	n	%	χ2	*p* Value
Age (years)										
18–28	268	28.2	195	72.8	5.53	0.063	95	35.4	3.43	0.1803
29–33	420	44.2	333	79.3			178	42.4		
>33	263	27.7	191	72.6			101	38.4		
Sex										
Male	243	25.6	174	71.6	2.83	0.093	92	37.9	0.22	0.6409
Female	708	74.5	545	77.0			282	39.8		
Marital status					-	0.768 ‖			0.00	1.000
Married	936	98.4	708	75.6			368	39.3		
Others †	15	1.6	11	73.3			6	40.0		
Educational level					31.37	<0.001			9.25	0.02618
Junior high school or below	62	6.5	34	54.8			19	30.6		
High school	143	15.0	92	64.3			43	30.1		
Bachelor’s degree	634	66.7	508	80.1			263	41.5		
Postgraduate degree or above	112	11.8	85	75.9			49	43.8		
Annual family income (CNY)										
≤50,000	49	5.2	29	59.2	30.41	<0.001	16	32.7	1.43	0.8382
50,001–100,000	175	18.4	110	62.9			66	37.7		
100,001–200,000	315	33.1	247	78.4			128	40.6		
200,001–300,000	228	24.0	187	82.0			92	40.4		
>300,000	184	19.4	146	79.4			72	39.1		
Relationship to the Child										
Mother	682	71.7	522	76.5	-	0.308 ‖	275	40.3	1.24	0.5392
Father	250	26.3	181	72.4			93	37.2		
Other	19	2.0	16	84.2			6	31.6		
Knowledge score ‡					132.64	<0.001			15.99	<0.001
Low	304	32.0	60	41.4			70	28.7		
Moderate	195	20.5	165	70.2			86	41.1		
High	452	47.5	494	86.5			218	43.8		
Total	951	100.0	719	75.6			951	100.0	374	39.3

† “Others” included unmarried, widowed, and divorced respondents. ‡ Scores represented the levels of knowledge regarding rotavirus and influenza vaccines among respondents. § Defined as having received at least one dose of the rotavirus vaccine. ‖ Calculated using Fisher’s exact test.

**Table 3:** Correct response rates for knowledge items regarding rotavirus and influenza vaccinations.

Vaccine	Knowledge Items	Correct Response Rate (%)
Rotavirus vaccine in children	Duration of vaccine-induced immunity	54.4
	Exposure to health education	61.1
	Sex disparity on susceptibility to rotavirus	61.5
	Modes of rotavirus transmission	66.1
	Rotavirus vaccine effectiveness	70.2
	Clinical manifestations of rotavirus infection	75.1
	High-risk populations for rotavirus infection	77.5
	Optimal prevention strategies	83.6
	General awareness of rotavirus vaccine	88.5
Influenza vaccine in parents	Distinction between influenza and common cold	50.0
	Necessity of annual influenza vaccination	63.6
	Typical clinical manifestations of influenza infection	68.9
	Influenza vaccine effectiveness	74.7
	Modes of influenza transmission	85.5
	High-risk populations for severe complications	88.4
	General awareness of influenza vaccine	92.4

**Table 4:** Correlations between the Behavioral and Social Drivers of Vaccination domains for childhood rotavirus vaccination and parental influenza vaccination.

		Childhood Rotavirus Vaccination			
		*Thinking and Feeling*	*Social Processes*	*Practical Issues*	*Motivation*
Parental influenza vaccination	*Thinking and feeling*	0.82	0.51	0.67	0.22
	*Social processes*	0.61	0.71	0.40	0.11
	*Practical issues*	0.65	0.30	0.84	0.14
	*Motivation*	0.24	0.09	0.15	0.21

## Data Availability

The datasets used and/or analyzed during the current study are available from the corresponding author on reasonable request.

## Supplementary Materials

The following supporting information can be downloaded at https://www.mdpi.com/article/10.3390/vaccines14040340/s1. Supplementary Material S1: Questionnaire used in this study.

## Abbreviations

The following abbreviations are used in this manuscript:

BeSD	Behavioural and Social Drivers
CDC	Center for Disease Control and Prevention
CFA	Confirmatory Factor Analysis
HPV	Human Papillomavirus
IQR	Interquartile Range
KMO	Kaiser–Meyer–Olkin
NIP	National Immunization Program
POVs	Points of Vaccination
SEM	Structural Equation Model
