# Supplementary material for: Parental Decision-Making for Themselves and Their Children in a Metropolis of China: Comparing Influenza and Rotavirus Vaccination Under the Behavioral and Social Drivers Framework
Source: Vaccines (Basel). 2026 Apr 12;14(4):340. doi: 10.3390/vaccines14040340 (PMC13119881; doi:10.3390/vaccines14040340)
Supplement: Supplementary file 1 [file vaccines-14-00340-s001.zip › vaccines-4232781-supplementary.pdf]

Survey on Behavioral and Social Drivers of Rotavirus and Influenza Vaccination

(original questionnaire in Chinese)

Section A. Basic Information

A1. District of residence: ________________

A2. Community health center where your child is receiving this vaccination:____

A3. Your date of birth:___________________

A4. What is your sex?

☐ Female

☐ Male

A5. What is your marital status?

☐ Single

☐ Married

☐ Divorced

☐ Widowed

A6. What is your highest educational attainment?

☐ Junior school or below

☐ High school

☐ Bachelor’s degree

☐ Postgraduate degree or above

A7. What is your annual household income (in CNY)?

☐≤50,000

☐50,001–100,000

☐100,001–200,000

☐200,001–300,000

☐>300,000

A8. Are you the child’s legal guardian or primary caregiver?

☐Yes

☐No (You may not be eligible to participate in this survey. The questionnaire ends

here.)

A9. What is your relationship to the child?

☐ Father

☐ Mother

☐ Grandparent

☐ Other (please specify): _________

Section B. Knowledge of Rotavirus and Rotavirus Vaccination

B1. Does the susceptibility to rotavirus infection differ by sex?

☐ Yes

☐ No

☐ Not sure

B2. Which population is at highest risk for rotavirus infection?

☐ Children aged 5 years and under

☐ Children and adolescents aged 5–18 years

☐ Adults aged 18 years and above

☐ Not sure

B3. What is the primary mode of rotavirus transmission?

☐ Fecal-oral transmission

☐ Droplet transmission

☐ Bloodborne transmission

☐ Vector-borne transmission

☐ Not sure

B4. What are the typical clinical manifestations of rotavirus infection?

☐ Diarrhea, cough, and runny nose

☐ Diarrhea, vomiting, and fever

☐ Diarrhea and skin rash

☐ Not sure

B5. What is the most effective strategy for preventing rotavirus infection?

☐ Appropriate introduction of complementary foods

☐ Wearing a mask

☐ Vaccination against rotavirus

☐ Breastfeeding

☐ Not sure

B6. Have you ever received health education related to rotavirus?

☐ Yes

☐ No

B7. Have you heard of rotavirus vaccine?

☐ Yes

☐ No

B8. What is the effectiveness of rotavirus vaccine?

☐ Less than 30%

☐ 50%-90%

☐ 100%

☐ Not sure

B9. How long does vaccine-induced immunity from rotavirus vaccine last?

☐ 1-3 years

☐ 10-15 years

☐ Lifelong protection

☐ Not sure

Section C. Child Rotavirus Vaccination Survey

C1. How important do you think rotavirus vaccination is for your child's health?

☐ Very important

☐ Moderately important

☐ Not very important

☐ Not important at all

C2. How safe do you think rotavirus vaccine is for your child?

☐ Very safe

☐ Moderately safe

☐ Not very safe

☐ Not safe at all

C3. How much do you trust the healthcare practitioners who administer the rotavirus vaccine to your child?

☐ Very much

☐ Moderately

☐ Not very

☐ Do not trust at all

C4. Do your family members support vaccinating your child against rotavirus?

☐ Yes

☐ No

C5. Have your relatives, friends, or colleagues recommended that your child receive the rotavirus vaccine?

☐ Yes

☐ No

C6. Have the local government or public health (CDC) authorities recommended that your child receive the rotavirus vaccine?

☐ Yes

☐ No

C7. Have the local government or public health (CDC) authorities contacted you to remind you when your child became eligible for rotavirus vaccine?

☐ Yes

☐ No

C8. What is your willingness of accepting rotavirus vaccine for your child? (1 = Completely unwilling; 5 = Completely willing)

☐ 1

☐ 2

☐ 3

☐ 4

☐ 5

C9. Has your child received the rotavirus vaccine so far?

☐ Has not received

☐ Has received some doses; next dose is not yet due

☐ Has received some doses; but not plan to complete remaining doses

☐ Has completed the full vaccination schedule

C10. Which type of rotavirus vaccine has your child received?

☐ Domestic monovalent rotavirus vaccine

☐ Domestic trivalent rotavirus vaccine

☐ Imported pentavalent rotavirus vaccine

☐ Not vaccinated

C11. Do you intend to vaccinate your child against rotavirus? (For parents whose children have not yet received the rotavirus vaccine)

☐ Yes

☐ No

C12. Do you know where to take your child to receive the rotavirus vaccine?

☐ Yes

☐ No

C13. Has your child ever been denied vaccination services due to unscheduled visit?

☐ Yes

☐ No

C14. How convenient is it for your child to receive the rotavirus vaccine?

☐ Very convenient

☐ Moderately convenient

☐ Not very convenient

☐ Not convenient at all

C15. Are you able to afford the cost of rotavirus vaccination? (Considering vaccine

cost, transportation expenses, loss of wages, etc.)

☐ Very affordable

☐ Moderately affordable

☐ Not very affordable

☐ Not affordable at all

C16. How satisfied are you with the rotavirus vaccination services?

☐ Very satisfied

☐ Moderately satisfied

☐ Not very satisfied

☐ Not satisfied at all

Section D. Knowledge of Influenza and Influenza Vaccination

D1. Is there any difference between influenza and common cold?

☐ Yes

☐ No

☐ Not sure

D2. What is the primary mode of influenza transmission?

☐ Fecal-oral transmission

☐ Droplet transmission

☐ Bloodborne transmission

☐ Vector-borne transmission

☐ Not sure

D3. What are the typical clinical manifestations of influenza infection?

☐ Fever, cough, and generalized body aches

☐ Diarrhea, vomiting, and fever

☐ Diarrhea and skin rash

☐ Not sure

D4. Which populations are at higher risk of developing severe complications from influenza?

☐ The elderly, children, pregnant women, or individuals with underlying diseases

☐ Healthy adults

☐ Not sure

D5. Have you heard of the influenza vaccine?

☐ Yes

☐ No

D6. What is the primary effect of influenza vaccination?

☐ To prevent influenza and its complications

☐ To treat influenza after infection

☐ To strengthen the immune system and prevent all diseases

☐ Not sure

D7. Is annual influenza vaccination necessary?

☐ Yes

☐ No

Section E. Adult Influenza Vaccination Survey

E1. How important do you think influenza vaccination is for your own health?

☐ Very important

☐ Moderately important

☐ Not very important

☐ Not important at all

E2. How safe do you think influenza vaccine is for yourself?

☐ Very safe

☐ Moderately safe

☐ Not very safe

☐ Not safe at all

E3. How much do you trust the healthcare practitioners who administer the influenza vaccine?

☐ Very much

☐ Moderately

☐ Not very

☐ Do not trust at all

E4. Do your family members support you receiving the influenza vaccine?

☐ Yes

☐ No

E5. Have your relatives, friends, or colleagues recommended that you receive the influenza vaccine?

☐ Yes

☐ No

E6. Have the local government or public health (CDC) authorities recommended that you receive the influenza vaccine?

☐ Yes

☐ No

E7. Have the local government or public health (CDC) authorities contacted you to remind you to receive the influenza vaccine during the influenza season?

☐ Yes

☐ No

E8. What is your willingness of accepting the influenza vaccine? (1 = Completely unwilling; 5 = Completely willing)

☐ 1

☐ 2

☐ 3

☐ 4

☐ 5

E9. Have you received the influenza vaccine for the 2024–2025 influenza season (since September 2024)?

☐ Yes

☐ No

E10. Do you know where to receive the influenza vaccine?

☐ Yes

☐ No

E11. Have you ever been denied vaccination services due to unscheduled visit?

☐ Yes

☐ No

E12. How convenient is it for you to receive the influenza vaccine?

☐ Very convenient

☐ Moderately convenient

☐ Not very convenient

☐ Not convenient at all

E13. Are you able to afford the cost of influenza vaccination? (Considering vaccine cost, transportation expenses, loss of wages, etc.)

☐ Very affordable

☐ Moderately affordable

☐ Not very affordable

☐ Not affordable at all

E14. How satisfied are you with the influenza vaccination services?

☐ Very satisfied

☐ Moderately satisfied

☐ Not very satisfied

☐ Not satisfied at all
